# NPPD: A Protein-Protein Docking Scoring Function Based on Dyadic Differences in Networks of Hydrophobic and Hydrophilic Amino Acid Residues

**DOI:** 10.3390/biology4020282

**Published:** 2015-03-24

**Authors:** Edward S. C. Shih, Ming-Jing Hwang

**Affiliations:** Institute of Biomedical Sciences, Academia Sinica, Nankang, Taipei 115, Taiwan; E-Mail: shihds@gate.sinica.edu.tw

**Keywords:** protein-protein docking, scoring function, dyadicity, amino acid network

## Abstract

Protein-protein docking (PPD) predictions usually rely on the use of a scoring function to rank docking models generated by exhaustive sampling. To rank good models higher than bad ones, a large number of scoring functions have been developed and evaluated, but the methods used for the computation of PPD predictions remain largely unsatisfactory. Here, we report a network-based PPD scoring function, the NPPD, in which the network consists of two types of network nodes, one for hydrophobic and the other for hydrophilic amino acid residues, and the nodes are connected when the residues they represent are within a certain contact distance. We showed that network parameters that compute dyadic interactions and those that compute heterophilic interactions of the amino acid networks thus constructed allowed NPPD to perform well in a benchmark evaluation of 115 PPD scoring functions, most of which, unlike NPPD, are based on some sort of protein-protein interaction energy. We also showed that NPPD was highly complementary to these energy-based scoring functions, suggesting that the combined use of conventional scoring functions and NPPD might significantly improve the accuracy of current PPD predictions.

## 1. Introduction

Living cells are a crowded environment in which most proteins interact with other proteins to exert cellular functions. To understand how protein-protein interactions mediate cellular processes, scientists often need to describe the structures of protein complexes at the atomic level. However, due to the difficulty in determining the atomic structures of protein complexes using experimental methods, protein-protein docking (PPD), a computational approach, is often used to complement results from experimental studies [1].

Most methods for PPD predictions involve a two-step strategy, sampling and scoring. For sampling, numerous docking models, also referred to as docking poses or decoys, are often generated from a global search of all possible relative orientations of, and separations between, two proteins that are brought together to form a complex, then these docking poses are ranked by a scoring function. To evaluate the performance of a given scoring function for a set of protein complexes, the TopN success rate is usually employed, in which a “success” hit for a complex is defined as when at least one of its top N docking poses, as ranked by the scoring function, satisfies a specified criterion for being a good (*i.e.*, near-native) model. It follows that, for a given scoring function, a higher success rate (*i.e.*, a higher number of correctly predicted complexes) can be obtained by choosing to compute the success rate at a larger N, since, for a given complex, there will be more poses and, thus, a higher probability of at least one being considered good. The objective when developing a good PPD scoring function is, therefore, to rank good poses as high and bad poses as low. However, despite significant progress in recent years, this is still an active area of research [2,3], as success rates are still low when small values of N are used (e.g., using a stringent criterion, Top1 and Top10 success rates are, respectively, generally below 10% and 20%), unless dockings are guided by experimentally-derived data or information [4,5].

Most PPD scoring functions use a set of mathematical equations to compute the energy resulting from the formation of the protein complex. To do so, many use molecular mechanics functions [6,7,8,9,10,11,12,13,14,15,16], while others use statistical mechanics methods to derive potentials from various sources, including experimentally-determined protein structures [8,10,17,18,19], docking decoys [6,20,21,22,23], homology models [24,25,26], or binding energy funnels [27,28]. Many non-energy-based PPD scoring functions have also been developed, including those that utilize bioinformatics-predicted information [29,30], shape complementarity [31,32], machine learning [33,34,35], coevolution [36], and amino acid networks (AANs) [37,38].

As described in the Experimental Section below, NPPD, the network-based PPD scoring function developed in this work, is based on AANs, which have also been referred to as residue contact networks [39], protein contact networks [40], protein structure networks [41], or residue interaction networks [42], although these networks may not be completely identical in terms of their construction (for reviews, see [39,40,41,43,44]). Owing to the appeal of network analysis in the era of post-genomics research, there has been an increase in the number of studies utilizing AANs to predict a protein’s functional sites [45,46,47], protein-protein [48,49,50,51] and protein-nucleic acid interaction [52,53], and to probe protein dynamics [42,54,55], folding [56,57,58] and structure [59,60,61,62,63]. Of these studies using AANs, two reports by Pons *et al.* [37] and Chang *et al.* [38] on PPD are directly relevant to the present work.

In AANs, the protein structure is modeled by a three-dimensional geometric network, with the amino acid residues (usually the Cα or Cβ atoms) being represented as network nodes and their contacts as network edges to capture the interactions between amino acids within the same protein structure and/or between two interacting proteins. Pons *et al.* [37] showed that network parameters, such as closeness and betweenness, can be used to suggest protein-protein interaction regions, and that an energy term that models this information can be added to an energy-based scoring function to improve PPD predictions. Chang *et al.* [38] used two networks for a single protein structure, one formed by hydrophobic residues and the other by hydrophilic residues, and analyzed the two networks from the same complex (docking pose) separately; their results again demonstrated that network properties can be used to assist conventional scoring functions to distinguish between good and bad PPD decoys.

Unlike Chang *et al.*, in developing NPPD, we constructed only a single network for a single protein structure, allowing both the hydrophobic (H) and hydrophilic (*i.e.*, polar, P) residue nodes to coexist in the same network. We were then able to investigate not only the effects of dyadicity calculated from the hydrophobic-hydrophobic (HH) and polar-polar (PP) interactions, but also the effects of heterophilicity calculated from the hydrophobic-polar (HP) interactions on the scoring of PPD poses. Benchmark evaluations showed that, using network parameters alone in all three methods, NPPD performed better than the network-assisted PPD predictions reported by Pons *et al.* [37] and Chang *et al.* [38], and that NPPD also performed well compared to most energy-based scoring functions. In addition, further analysis revealed significant complementarity between NPPD and the other scoring functions evaluated, demonstrating the merit of using a combination of NPPD and other types of scoring functions to further improve PPD predictions.

## 2. Experimental Section

**Figure 1:**
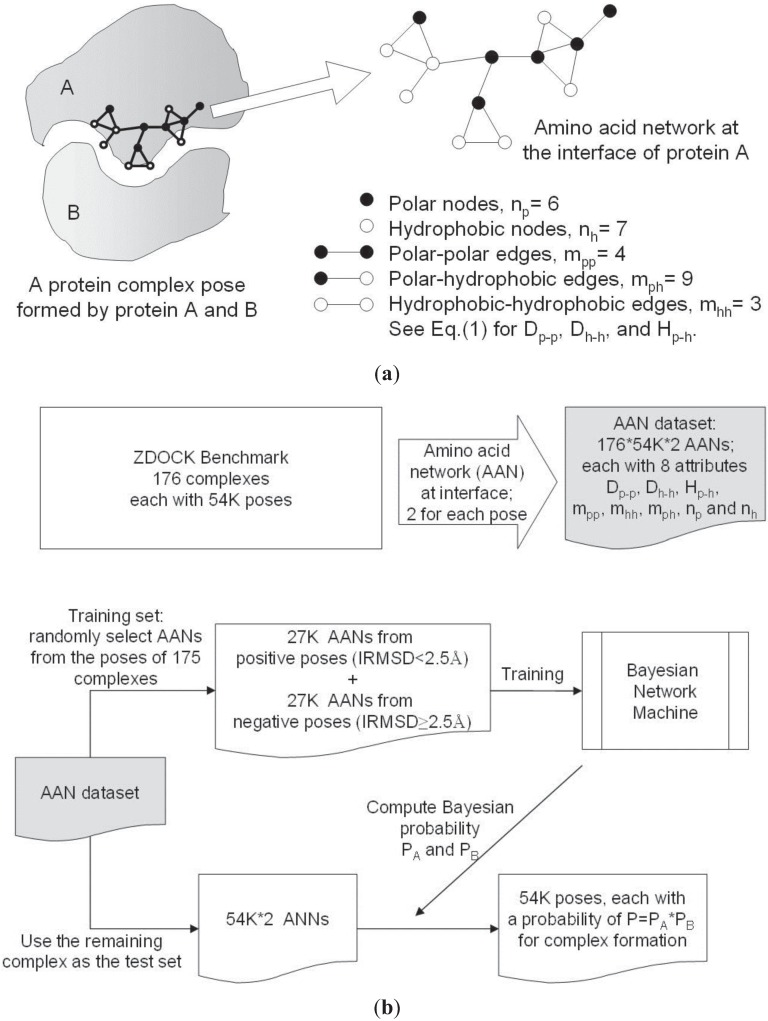
Procedures used to develop NPPD. (**a**) An example of an amino acid network and the network parameters used in this study for a docking pose; (**b**) Flowchart of the training and testing of a Bayesian network model of NPPD.

Figure 1 outlines the procedures used to develop NPPD. Briefly, the interface residues of a given complex (*i.e.*, docking pose) of protein A and protein B were determined, yielding the H and P nodes for the construction of the AANs for A and B. Eight parameters for each of the two networks were computed and served as attributes for training and testing a Bayesian network model using a PPD benchmark dataset. Note that, during the training of the Bayesian model, the complex context of all the poses was removed and each AAN was treated independently, although, during the machine learning, those that came from a good pose were used as positive incidences and those from a bad pose as negative incidences. Using the Bayesian model thus derived, NPPD can then score any given pose by multiplying together the Bayesian probabilities of the two AANs. This has the advantage of quickly eliminating most of the bad poses since it takes just one bad AAN (*i.e.*, a low Bayesian probability) to produce a bad product (pose) of two AANs. Note that, as illustrated in Figure 1, our AAN was constructed on one side of the interface and did not extend to include contacts from the other side, because including inter-protein contacts did not improve the results [64], possibly owing to the fact that the connections of an inter-protein network can change significantly even by minor changes in the configuration of the docking pose. Still, it may be warranted for future studies to find a way to use inter-protein contacts productively in the Bayesian model.

### 2.1. Docking Datasets, Poses, and Quality Measures

The 176 protein complexes used in this study were retrieved from a PPD benchmark dataset of known atomic structures of complex component proteins in both the bound (complex) and unbound (free) form [65]. For each of the 176 complexes, two sets of docking poses from the unbound form were used to evaluate the performance of NPPD and compare it with those of several other PPD scoring functions. One set contained the top 54,000 poses for each of 176 complexes generated by ZDOCK [66] and was downloaded from its website (http://zlab.umassmed.edu/zdock/decoys.shtml). The other set, kindly provided by the authors of a large-scale evaluation of 115 scoring functions [67], consisted of ~500 poses generated using SwarmDock [68] for each of a subset containing 118 complexes. The two sets came with their own quality measures for near-native poses, *i.e.*, the so-called good poses; that used for the ZDOCK-generated set was an interface RMSD (IRMSD) < 2.5 Å, where IRMSD is the root mean square displacement of the interface residue’s Cα atoms from the experimentally determined structure of the bound complex and an interface residue is defined as one having at least one heavy (non-hydrogen) atom within 5 Å of any heavy atom in the second protein of the complex, while those used for the SwarmDock-generated set were three quality measures from the CAPRI criteria [2] for acceptable, medium, and high quality.

### 2.2. Amino Acid Networks and Network Parameters

As described above, two AANs were constructed from the interface residues of two interacting proteins locked in a docking pose. In this work, the 20 amino acids were divided into two classes according to Eisenberg *et al.* [69], the H class consisting of Gly, Ile, Leu, Val, Phe, Met, Trp, Cys, Tyr, and Ala, and the P class consisting of Lys, Thr, Ser, Gln, Asn, Glu, Asp, Arg, His, and Pro. Our AANs, thus, contained two types of nodes, H and P, and a network edge was established to connect any two nodes (residues) if any heavy atom in one of the residues was within 5.0 Å of any heavy atom in the other (Figure 1a).

For each AAN, we computed two dyadicity parameters, D_p-p_ and D_h-h_, and one heterophilicity parameter, H_p-h_, which, following the work of Park and Barabasi [70], are defined as:

(1) $$D_{pp}\equiv \frac{m_{pp}}{E(m_{pp})},D_{hh}\equiv \frac{m_{hh}}{E(m_{hh})},\;\text{and}\;H_{ph}\equiv \frac{m_{ph}}{E(m_{ph})}$$

where *m_pp_*, *m_hh_*, and *m_ph_* are, respectively, the number of P-P, H-H, and P-H edges in the AAN, and the three denominators are the respectively expected values of *m_pp_*, *m_hh_*, and *m_ph_*, which can be computed as:

(2) $$E(m_{pp})=\frac{n_{p}(n_{p}-1)}{2}p,\;E(m_{hh})=\frac{n_{h}(n_{h}-1)}{2}p\;\text{and}\;E(m_{ph})=n_{p}n_{h}p$$

where *n_p_* is the number of P nodes, *n_h_* the number of H nodes, and *p* = 2M/N(N-1) (M and N are the total number of edges and nodes, respectively) is connectance, which represents the average probability that two nodes in a dyadic network are connected [71].

### 2.3. Bayesian Network

To infer whether two AANs would generate a near-correct docking pose, we employed the machine learning algorithm implemented in the Weka platform [72] to derive a Bayesian network model [73], which we then used to compute the probability for every AAN of being at the interface of a protein complex. We then computed the probability product of two AANs to give an estimate of the likelihood of the resulting docking pose being a good one (Figure 1b). The aforementioned 176 benchmark complexes and their 54,000 poses per complex generated by ZDOCK were used in a leave-one-out training and testing of the Bayesian model, *i.e.*, each of the 176 complexes was, in turn, left out during training of the model on AANs randomly selected from poses of the remaining 175 complexes and was then used as a test case. As shown in Figure 1b, we randomly selected 27,000 AANs from good poses, irrespective of whether they came from the same complex or not, as positive incidences and an equal number of AANs from bad poses as negative incidences, and used the values of the 8 parameters of D_p-p_, D_h-h_, H_p-h_, m_pp_, m_hh_, m_ph_, n_p_ and n_h_ of the AANs as attributes for training. The training set-derived Bayesian model was then used to score poses of the left-out complex as a test of the model.

## 3. Results and Discussion

### 3.1. Performance of NPPD and IRAD

**Figure 2:**
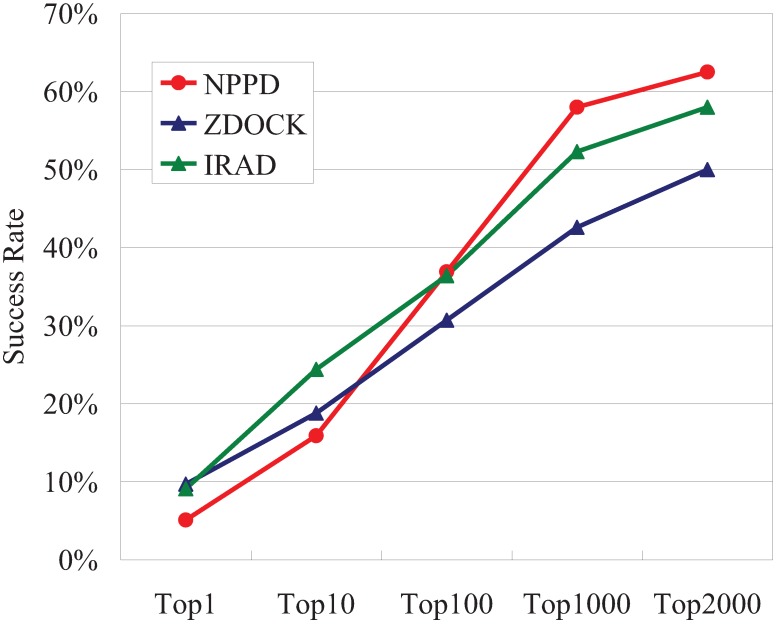
TopN success rates for NPPD, ZDOCK, and IRAD on the benchmark dataset of the unbound docking poses of 176 protein complexes. IRMSD < 2.5 Å was used to determine good (near-correct) poses. The success rates of ZDOCK and IRAD were obtained from the ZDOCK website (http://zlab.umassmed.edu/zdock/perf_decoys.shtml).

The TopN success rates obtained using poses created and ranked by ZDOCK [66] and IRAD [74], a state-of-the-art PPD scoring function, have often been used as yardsticks to evaluate PPD scoring functions [3,4,5]. Both ZDOCK and IRAD use a multitude of scoring terms, such as shape complementarity, interface atomic contact energy, and electrostatics, and IRAD also uses both atom-based and residue-based potentials [66,74]. As can be seen in Figure 2, using the 54,000 poses created by ZDOCK for each of the 176 benchmark complexes, the Bayesian probabilities of NPPD produced worse Top1 and Top10 success rates than either ZDOCK or IRAD, but, as N increased, the success rates increased faster for NPPD than for ZDOCK or IRAD, with NPPD outperforming the other two when *N* > 100.

Despite the low success rates of NPPD at a low N, it is interesting that, as shown in Table 1, many of the complexes that NPPD succeeded at predicting were different from those predicted by IRAD and vice versa. The complementarity between the two methods, measured as the ratio of the method-unique successes divided by all successes and expressed as a percentage, was especially significant at low N, being as high as 86% for the Top1 success rate (only 3 out of 22 complexes were successfully predicted by both methods).

**Table 1:** Number of benchmark complexes successfully predicted by NPPD and/or IRAD at different TopN success rates.

Set	Top1	Top10	Top100	Top1000	Top2000
NPPD (A)	9	28	65	102	110
IRAD (B)	16	43	64	92	102
Intersection (A∩B)	3	15	44	80	95
Union (A∪B) = a	22	56	85	114	117
Unique to NPPD or IRAD (A⊖B) = b	19	41	41	34	22
Complementarity = b/a	86%	73%	48%	30%	19%

⊖ (Symmetric difference): the set of elements in either of the sets and not in their intersection.

### 3.2. Comparison with Other Network-Based Methods

As mentioned in the Introduction, two other groups have used AANs to help score docking poses [37,38]. Table 2 compares our results with their reported success rates and shows that, using the same benchmark dataset and the same criterion for success hits, when the scoring was based on network parameters alone, NPPD produced a better Top1 and Top10 success rate: e.g., the values for the Top10 success rate was 18.5% using NPPD *versus* 10.6% in Pons *et al.* [37] for the 176 complexes of the benchmark and 25.6% using NPPD *versus* 23.2% in Chang *et al.* [38] for a subset of 43 complexes. However, it should be noted that different sampling algorithms (FTDOCK [16], RossettaDock [75], and ZDOCK [66]) were used to generate the same number of poses for evaluation, which may have contributed to the differences in success rates obtained. Several aspects of the use of AANs were also different: (i) as mentioned earlier, our AAN was different from that of Pons *et al.* [37], which represents all amino acids by just one type of network node, and from that of Chang *et al.* [38], which, although, like ours, has both H and P nodes, creates two separate AANs for the two different types of nodes; (ii) as also mentioned earlier, unlike these two other networks, our AAN did not include inter-protein contacts; (iii) whereas we used dyadicity and heterophilicity parameters for scoring, the other two studies used more conventional network parameters, such as degree and cluster coefficient [38] and closeness and betweenness [37]; (iv) NPPD was used to score docking poses by itself, whereas the network-based scoring functions of the other two studies are additional terms that can be added to an existing scoring function to give a better result [37,38] (Table 2), and, if these results also apply to our method, incorporating NPPD into existing scoring functions should achieve significantly higher success rates.

**Table 2:** Conditions and Top1/Top10 success rates for NPPD and two other network-based scoring functions.

**Conditions of docking poses**	**176 Complexes**		**43 Complexes**	
	Pons *et al.* [37]	NPPD	Chang *et al.* [38]	NPPD
Generation of docking poses	FTDock [16]	ZDOCK	RossettaDock 1.0 [75]	ZDOCK
Number of poses generated	10,000		1000	
Criterion for a success hit	L-RMSD < 10 Å		L-RMSD < 5 Å	
Top 1 success rate *	5.0% (7.0%)	8.0%	2.3% (25.6%)	11.6%
Top10 success rate *	10.6% (29.8%)	18.5%	23.2% (53.4%)	25.6%

* The values in parenthesis are success rates produced by combining the network parameters and the energy terms of the sampling method.

### 3.3. Performance of NPPD in a Comprehensive Evaluation of a Number of PPD Scoring Functions

**Figure 3:**
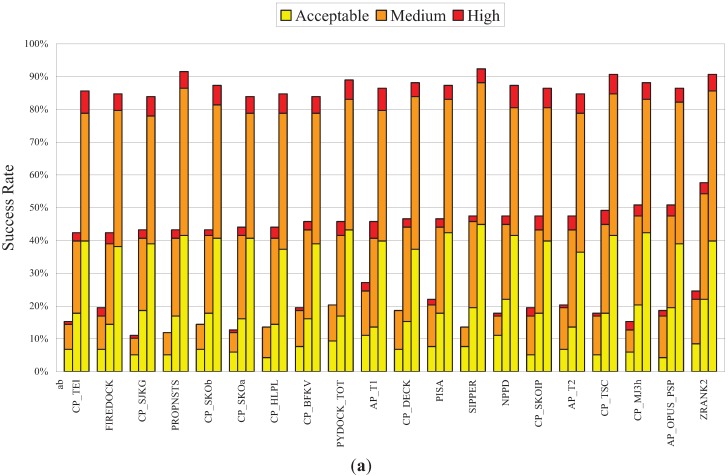

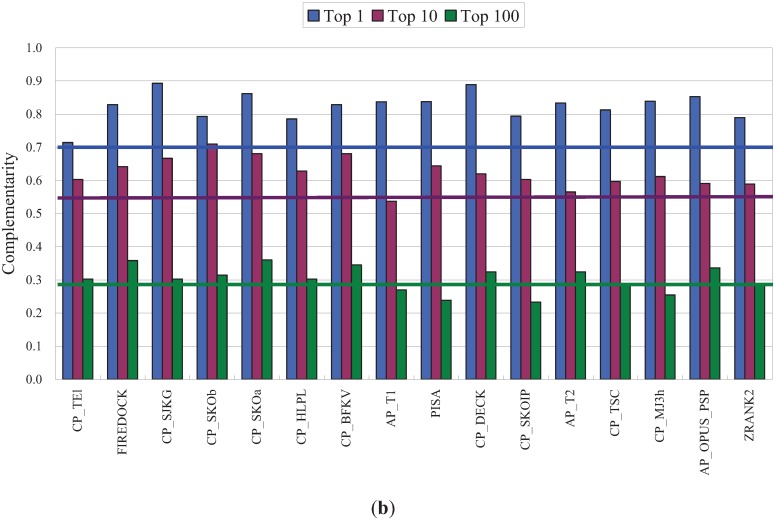
Benchmark results for NPPD and complementarity of NPPD and several best performing PPD scoring functions. (**a**) The 20 best performing PPD scoring functions ordered, from left to right, by increasing Top10 success rate. All data except those for NPPD were taken from [67]. Note that the Top1, Top10, and Top100 success rates for each method, shown, respectively, as the left, center, and right bar in each group, were computed using a set of unbound docking poses (~500 for each of 118 complexes) generated by SwarmDock [68], which was different from the set generated by ZDOCK used in Figure 2 and Table 1. The leave-one-out Bayesian model of NPPD was therefore derived using these SwarmDock poses, but otherwise using the same procedures described in Figure 1. The portions of success rates for high, medium, and acceptable quality poses are shown, respectively, in red, orange, and yellow, the criteria for the three quality measures being those used by CAPRI [2]; (**b**) Complementarity between NPPD and each of another 16 best performing PPD scoring functions. The blue, purple, and green bars indicate the complementarity, as defined in Table 1, computed based on, respectively, the Top1, Top10, or Top100 success rates. The horizontal blue, purple, and green lines are the averaged complementarity for, respectively, theTop1, Top10, or Top100 success rates for all pairs of the 16 scoring functions (three of the scoring functions (SIPPER, PYDOCK_TOT, and PROPNSTS) of the 19 compared in (**a**) were not included because the data were not made available to us). References for these 19 PPD scoring functions can be found in Reference [67] and references therein.

Since many factors can affect the performance of PPD scoring functions, one example being the evaluation of docking poses produced by different sampling methods as mentioned above, it was important to evaluate NPPD further. Recently, a large-scale evaluation of 115 PPD scoring functions was reported [67], in which the authors ranked these scoring functions by comparing their Top1, Top10, and Top100 success rates on a set of docking poses produced by SwarmDock [68]. As shown in Figure 3a, using the same set of docking poses, the leave-one-out Bayesian model of NPPD produced TopN success rates comparable to those produced by the best performers of the 115 scoring functions evaluated (ranked 7th by Top10 success rate). Note that, with the exception of the 1^st^-ranked ZRANK2 method [12], an earlier version of IRAD, which perhaps stands out a little bit from the others, these 20 top performers were more or less equally good, as the absolute ranking depended on which success rate (Top1, 10, or 100) and which quality measure (acceptable, medium, or high) were used as the basis for ranking. Note also that, of these top performers, NPPD was the only one using network parameters (the scoring functions of Pons *et al.* [37] and Chang *et al.* [38] were not included in the 115 PPD scoring functions previously evaluated [67]).

Using the complementarity between two PPD scoring functions as defined in Table 1, *i.e.*, the ratio of the number of complexes successfully predicted by either, but not both, of the two functions divided by the total number of successfully predicted complexes, the results, presented in Figure 3b, showed that the complementarity of NPPD with each of 16 other best performers was generally higher than the averaged complementarity exhibited by the other methods, especially in the case of the Top1 and Top10 success rates. Interestingly, although SPIDER [76], another AAN-based PPD scoring function, ranked only 38th of the 115 scoring functions evaluated [67], it is especially good at predicting complexes not detected by conventional scoring functions [67]. Unlike NPPD and the methods used by Pons *et al.* [37] and Chang *et al.* [38], SPIDER uses motifs of network structures, rather than network parameters, for scoring.

### 3.4. Some Limitations and Prospects

**Figure 4:**
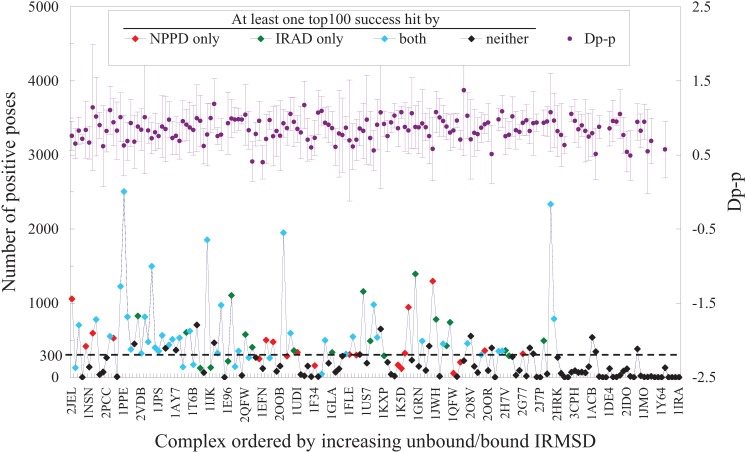
Number of positive poses and D_p-p_ plotted against unbound/bound IRMSD. The 176 benchmark complexes of ZDOCK are ordered in increasing unbound/bound IRMSD, the best RMSD of interface residues superimposed between the unbound form and the bound form of the complex, with the PDB ID of every 5^th^ complex indicated on the X-axis. Dashed line denotes a number of 300 positive poses. In the top half of the figure are the averages and standard deviations of the parameter D_p-p_ computed from the positive poses of each complex; all other attributes used by NPPD, and for negative poses, showed a similar random distribution [64].

Without the ability to handle large conformational change induced by complex formation, PPD methods would perform badly for such complexes [2]. Indeed, both NPPD and IRAD failed to produce a Top100 success hit for those in the benchmark set with the largest unbound/bound IRMSDs, indicative of a significant change in conformation between the unbound and bound form of the complex (Figure 4). However, conformational change is not the only culprit for failures in PPD predictions. Figure 4 shows that if sampling could not produce a sufficient number, say 300, of positive (good) poses as defined by IRMSD < 2.5 Å (see Figure 1b) to score upon, the likelihood for either NPPD or IRAD to succeed was drastically decreased, even for complexes considered as “rigid” [65]. Further analysis indicated that some of these “rigid” complexes had a particularly small interface and hence might be difficult to sample and predict [77]. Since the best current scoring functions all performed similarly (Figure 3), we speculate that the same two factors, conformational change and insufficient sampling of good poses, also limit the success of other PPD methods. Note that while the sampling of good poses among different complexes was unbalanced, the distribution of the attributes used by NPPD was not (Figure 4), suggesting that sampling bias would not significantly affect training of the Bayesian model. While it is not entirely clear to us what gave rise to the apparently poor correlation between the number of good poses sampled and unbound/bound IRMSD as observed in Figure 4, it is notable that NPPD was better than IRAD for a few of those with the smallest unbound/bound IRMSDs and poor sampling, whereas IRAD did much better than NPPD for those ranked next in unbound/bound IRMSD (roughly between complex 1PPE and 2QFW in Figure 4), thereby contributing partly to the high complementarity between the two methods (Table 1). Taken all these results together, we can conclude that while it is still likely to significantly improve PPD performance by combining all the different scoring functions, the main barriers to overcome remain those arising from sampling and conformational change.

In this work, instead of using two-fold validation as did Chang *et al.* [38], we opted for the leave-one-out validation of machine learning so that every complex of the benchmark set can be a test and the performance of NPPD can be fully compared with other scoring functions. Technical differences aside, machine learning techniques are known to be unreliable for extrapolation, and only methods based on first-principles physics can truly predict and would not fail miserably when encountering complexes with an unusual interface [78]. However, as such an ideal method is not yet in sight, there is room and merit to further develop empirical methods, such as NPPD, since a new method, particularly a nonconventional one, can often reveal shortfalls of existing methods.

## 4. Conclusions

In this work, we showed that a Bayesian model based on the dyadic parameters of AANs of docking poses performed well compared to the best scoring functions currently used for PPD predictions. Furthermore, the results showed that our method can complement other methods by finding good poses for a significant number of complexes missed by these methods. Taken together with the findings in a recent large-scale evaluation of 115 PPD scoring functions [67], these results suggest that non-conventional scoring functions, such as that developed in the present study, are worthy of further investigation in the effort to improve the prediction of protein complex structures.
